# Indents

**Published:** 1917-04

**Authors:** 


### INDENTS

**☞ Brief Articles of Technical or Scientific Value will receive notice and comment. Contributions invited.**

*Mouth Wash* To the Editor:—In the *Journal of Infectious Diseases,* 1906, 3, 793, there was an article by Dr. A. B. Wadsworth, now director of the Division of Laboratories and Research of the New York State Department of Health, giving a detailed description of a mouth wash. Please publish it in Queries and Minor Notes.—H. D. T.

Answer:—

| | gm. or c.c. | | |
|---|---|---|---|
| Sodium chlorid ........ | 2 | | ʒ ss. |
| Sodium bicarbonate .... | | 7 | gr. x |
| Water (dist.) ......... | 62 | | ℥ ii. |
| Glycerin ............... | 31 | | ℥ i |
| Alcohol ................ | 155 | | ℥ v |
| Thymol ................ | | | |
| Menthol .............āā | | 07 | gr. i |
| Oil of wintergreen....... | | 18 | gtt. iii |
| Oil of cinnamon......... | | 12 | gtt. ii |
| Oil of eucalptus......... | | 32 | gtt. v |
| Tr. cudbear............ | 6 | | ʒ iss. |
| Tr. rhatany ............ | 2 | | ʒ ss. |

Dissolve salts in water before adding alcohol. For use add an equal part of water.—*Journal A. M. A.*

*Noncorrosive Broach* To make a broach for iodine applications cut a strip of aluminum plate about two inches long and about one-sixteenth of an inch wide, or make it tapering from the part to be held in the fingers to a point. The corners may be filed round and the handle end inserted in a split stick if a long handle is required. It can also be set in the handle at any desired angle, or used in a broach holder. Wrap a little cotton on the applicator end and it will carry medicines into pyorrhea pockets and elsewhere with great convenience. A larger wrapping of cotton makes it serve as a splendid swab for more liberal applications.—P. A. Pyper, in *Dental Review.*

*Relative Values* "Well, George," said the president of the company to old George, "how goes it?"

"Fair to middlin', sir," George answered. And he continued to currycomb a bay horse.

"Me an' this here hoss," George said suddenly, "has worked for your firm sixteen year."

"Well, well," said the president, thinking a little guiltily of George's salary. "And I suppose you are both pretty highly valued, George, eh?"

"H'm," said George, "the both of us was took sick last week, and they got a doctor for the hoss, but they just docked my pay."—*Home Companion.*

*Chicago's Dental Week* By order of the mayor of the city, a week beginning December 4, 1916, was devoted to the examination of the children's teeth. The profession of the city, the dental colleges and health authorities, all co-operated to make the research complete. So far as was practicable, advice and service were given, as well as dentifrice and tooth brushes, so far as the supply would permit. It is expected that further follow-up work will be done.

*Care of the Hands* In doing laboratory work which is likely to soil the hands, it is a good plan to wash the hands first and dry partially, and then anoint them completely with olive oil. When the work is completed the oil and stains are easily removed by washing in warm, soft water and soap.—J. M. H., in *Dental Review.*

*Bridgeport, Connecticut, School Clinic Report* The second annual report shows 20,850 prophylactic treatments to 10,990 children. 18,006 children received instruction in prophylaxis by the tooth brush demonstration method. Out of 1,946 children in the fifth grade, 1,867 had well defined malocclusion, 77 had clean teeth, 658 fairly clean teeth, 1,211 had dirty teeth—apparently the tooth brush drill missed the fourth grade last year, and the orthodontists of Bridgeport need help. 10,726 cavities were found in the permanent teeth. This means that every dentist in Bridgeport will have five children's teeth to fill every day for a month to clean up this condition alone, to give the school children a fair chance to make good the rest of the year, besides the extractions, cleanings and treatments needed. That looks like some job for a lot of already busy dentists.

*Use for Old Fissure Burs* There are many occasions for the use of a drill. Opening through enamel for cavity extension, gaining entrance on the lingual surface of an anterior tooth for pulp removal, cutting old amalgam and many other operations. Take an old fissure bur, grind it flat on two sides, leaving the two edges with the old cuttings on them. Then grind the point with its two bevels to suit your desire. The more the taper to this bevel the faster it will cut. This will make a better drill than you can buy.—*Pacific Gazette.*

*Plumpers for Vulcanite Plates* A good method consists in making a tube of vulcanizable rubber about one inch long, of the thickness of a lead pencil, putting a drop of water inside, and closing the ends of the tube securely; this is placed in the investment, and a little rubber packed around it. This will insure a hollow plumper, much lighter than a solid one, and free from porosity.—E. J. T., in *The Dental Cosmos.*

*Prevention of Caries* I venture to state with confidence that if every child born could have the proper mouth toilet administered by its mother or nurse until it is two years of age, and then a proper tooth brush toilet established and persistently maintained, that dental caries would be reduced from fifty to eighty per cent.—Dr. B. S. Sutherland, *Michigan Dental Journal.*

*Recementing a Loose Bridge Without Removing* When the gold crown abutment attachment of a large bridge becomes loose, drill a hole in the gold cap with a number seven bur, and wash out all the loose debris. Take a rubber cup, such as is used for polishing teeth that is mounted on a mandrel, and fill it with a slow setting cement. Then lift up the gold cap as much as possible and dry out. Place the cup filled with cement over the hole and force the cement into the space by a sliding motion. Repeat the operation until the cavity is full. Then place a finger over the hole to prevent the cement from coming out, and press the crown down into place. As soon as the cement has set the hole can be filled with a durable filling, if desired.—Dr. L. E. Custer, in *Dental Summary.*

*Why are Some Teeth Immune to Caries?* Dr. Flint of Pittsburg, in discussing a paper read by Dr. Grace Rogers Spalding before the joint meeting of the Orthodontists and Periodontists said that as a practitioner of orthodontia his greatest fear was, his work might fail in some important manner because of the general carelessness of patients in keeping the teeth on which he placed appliances clean. He said he could not account for the fact that one of the best sets of teeth he ever saw was in the mouth of a boy who kept the filthiest mouth he ever worked over. Dr. Spalding, in reply, quoted Dr. G. V. Black, who said he could not understand why he so often encountered third molar teeth that were practically covered with soft deposits and yet they showed not the slightest degree of caries. His only conclusion was that no respectable microorganism would deign to inhabit such an environment.

*Antidote for Novocain-Suprarenin* We have discovered that sulfuric ether is the best antidote for the toxic effects of this anesthetic. The experiments at the University of Minnesota determined that this anesthetic given to animals to the extent of producing convulsions brought about recovery by the administration of ether. Ether, therefore, becomes the approved antidote.—Dr. W. H. G. Logan, in *Dental Review.*

*Exemption from Army Service of a Dentist* The British Central Tribunal has granted exemption to a dentist on the grounds that he is the only dentist serving a large number of people, and it would be a serious menace to their health if he were to leave his practice. It was therefore expedient in the national interest that he remain engaged in his civil pursuit rather than to go to the front.—*Lancet.*

*Gold Crown Repair* A small hole or slit in a shell crown can be very easily and neatly repaired by the following method: Clean the crown in acids, adjust the parts to their correct relationship. Firmly engage it in a suitable pair of pliers that will not conduct away too much heat. Have handy several cylinders of gold foil and a pair of pliers to place them. Flux the part to be repaired and heat the work in the open flame to a dull red, while at this temperature the foil may be attached by simply bringing it in contact with the crown surface. As much foil may be applied as is deemed necessary for strength and contour, and when cooler may be burnished to form. Then flux again and flow a little solder of suitable carat over this foil. This will make a strong and neat repair.—Dr. F. W. Frahm, in *Pacific Gazette.*

*A Dentist's Duty to His Profession* When a man is graduated from a dental college and thereby assumes a professional status in the world he tacitly accepts a double responsibility. In addition to the duty which every man owes to his family and himself, as intimated in the consideration of business methods, there is a finer and I sometimes think a higher obligation devolving upon him in his attitude toward the profession and the public. To properly practice a profession means much self-sacrifice. The dollar should not be the dominant factor in professional life—important as the dollar is—and no man who permits the dollar to dominate his every action should assume to take rank as a truly professional man.—C. N. Johnson, in *Journal N. D. A.*

*Another German Imposture* The war has opened wide the eyes of the world to the dishonesty of Germans in general, and has shown the fallacy of regarding Germans as preeminent in science. It is true to state that Germans debase any science they touch, a good example being the introduction by their chemists of the use of poison gases into warfare. Dr. Charles A. Mercier in the *British Medical Journal* has a scathing exposure of the claims of Germans to have established a branch of mental science called psychoanalysis. Dr. Mercier sums up his exposure as follows:

"For more than twenty years I have been demonstrating the rottenness of German teaching in mental disease, and during all that time I have been as the voice of one crying in the wilderness. There is no folly so egregious, no sham so transparent, that the alienists of this country will not gulp it down with greedy credulity if only it comes from Germany. The superstition that Germany is preeminent in mental disease is as deeply rooted and as irrational as the superstitions that she is preeminent in organization and in science generally. The last are now exploded. The whole world is at length ashamed as having so long suffered itself to be imposed upon by braggart boasting. Even Americans, the most gullible people in the world, as they are the most generous in giving intellectual credit, are realizing that Kultur is a sham, and the miraculous German capacity for organization and for scientific discovery an imposture. It is left for the alienists of the country, and for a little tail of other physicians, to be the very last to cling to an exploded superstition.—*British Dental Journal.*

*Soldering Accident Repairs*

Sometimes during the process of soldering a Richmond or bridge, a portion of tne backing will burn exposing the porcelain. The repair of the damage without even removing the work from the fire or allowing it to cool down can be accomplished by patting cylinders of foil over the area until the desired contour or repair has been made. Then allow the solder to flow over and through this foil, care being observed in the use of the blow-pipe.—*Pacific Gazette.*

*New Terms*

Dr. Ottolengui, in the February *Items of Interest,* suggests some new terms for more definite meanings in radiology. He says: "It will save us much confusion when making a diagnosis from a radiograph if we might have a word having the limited and special significance which would register exactly what we mean and nothing else. The word apoque means 'impervious to light.' This solves our problem. We may coin the word 'radiopaque,' and it would definitely mean "impervious to the X-ray.' The construction likewise would be etymologically correct. Radiopaque, from the Latin, *radius*—a ray, plus *opacus*—a shade. From radiopaque we would likewise have the useful term radiopacity. We might also coin the word radioparent, and radioparency to indicate that quality of matter which offers no barrier to the X-ray. (Latin, *radius*, plus *pareo*), and the word radiolucent (likewise radiolucency), to mean offering slight resistance to the ray (Latin, *radius*, plus *luceo*).

*Dental Operations Concessive* Dr. E. C. Kirk remarks that after all, every dental operation we make necessarily is a compromise with disease conditions. If this is true, why do we speak of cures, radical surgery, restorations, etc.?

*Cellutone for Repairing Models* Dissolve fifteen grains of clear celluloid—such as is used for windows in automobiles and the like—in one ounce of acetone. With this solution a broken plaster cast may be repaired so perfectly that it is difficult to detect the place of fracture, and when dry the joint will be so secure that the cast will fracture at some other place than where the repair has been made if force enough is applied. —*Dental Review*.

*Cast Gold Dowels for Dowel Crowns* With the advent of cast gold work our methods in crownwork and bridgework have been wonderfully improved. We can now crown roots which ten years ago would have been condemned and we can also obtain a perfect fit with added strength. All this is due to the accurate and direct impression we take of the tooth to be restored. For many years I filled up the different forms of platinum wire supplied to us and tried to make the dowel fit the canal as accurately as possible, and wasted, in doing so, many shillings' worth of that valuable metal.

There were many opinions as to which was the most serviceable alloy of platinum to be used, but in my hands there were only two which gave satisfaction, and they were iridio-platinum and dental alloy wire. Another point hard to decide was the proper form to give dowels, as some men preferred a square pin, others a round or elliptical. Then there was the question of how far should a pin be carried up a root canal to give the requisite strength, many good bridgeworkers believing in short and thick pins. We can overcome all these doubts now if we cast a dowel to fit the root canal correctly. I suppose it is two years since I commenced using cast dowels for dowel crowns, and by their use I have discarded bands for strengthening purposes. I contend that the result one gets with a cast dowel depends upon the preparation of the root canal. The canal must be prepared to have a definite form according to the type of tooth, and I like the dowel to be carried two-thirds up the root canal, the canal to be given a good opening to allow easy withdrawal of the wax impression. My method of procedure is as follows: Given an upper central root, the labial surface is ground with a slight bevel from the center under the gum, and the lingual surface likewise. The canal being given a definite shape is finally finished off with pointed plug finishing burs and rounded at its entrance with small stones. A stick of modeling compound is then softened slightly at the top and gradual pressure is exerted upon the root as the compound hardens. This pushes back the gum from the circumference of the root. The impression wax is then softened, made conical and pressed up the canal, the surplus being pressed over and about the root. When hard, it is trimmed to the gum margin, a sprue inserted and withdrawn. In the case of fractured or split roots, the impression is withdrawn and trimmed to follow the contour of the part of the root to be restored. The wax on the lingual side, or the part of the root standing sound, is pressed under the gum and around the root. This gives the cast the same result as a half band. The gold cast being made, it is placed back in the root again and a plaster impression taken. Where an all-porcelain tube tooth is used, an impression of the ground crown in position in the wax can be taken of the whole cast and inserted. Before setting cast dowel crowns I make the dowels slightly rough with a fine wheel bur. I do so for the reason that in polishing the completed crown the dowel part often gets a high polish, with the result that the small amount of cement between the dentin and the dowel may not grip the dowel.

I omitted to mention that I have had the most satisfactory results with the use of coin gold or platinized gold for these casts.—Basil Jones, D.D.S., *The Commonwealth Dental Review.*

*Technic of Root Filling*

In filling large canals, especially those in connection with which dental granulomas, or alveolar abscesses, have been treated, where the apex is large, we ought not to expect to get response from the patients on account of the loss of tissue in the periapical area. It is best here to measure, with cotton on a broach, the approximate size of the canal and then use one cone which approximately fits the canal rather than use two or three smaller ones, with the possibility of unnecessarily forcing one through the apex and into the periapical area. With the approximate size of the canal in mind, the latter is moistened with chloroform and this followed with eucapercha. Then the proper sized cone, flattened on the large end, should be dipped in chloroform and placed in the canal, with the pliers. When the point is thus introduced, it should be worked up and down a few times with a slight pumping motion. In this manner the chloroform and the eucalyptol soften the outer surface of the cone, leaving the central portion with sufficient resistance to cause the guttapercha to spread laterally. The cone is then pressed to place and root canal pluggers may be used and the mass of gutta-percha packed in the canal. If chloroform is used, very little heat is required to soften the cone. Finally, the packing may be done with a tampon of cotton or Japanese bibulous paper, saturated with chloroform as suggested by Rhein.

In filling large canals from which live pulps have been removed, the patient will usually flinch when the cone is first introduced. When this occurs, we should wait a few seconds, when the cone may be gently pressed much farther without causing the patient to flinch a second time. If these precautions are observed, they will be the means of guarding against much of the pericementitis which frequently follows. In filling all root canals we should make every endeavor to have the sterile root filling reach the end of the root. Dr. Rhein has proposed encapsulating the apical end of the root with fluid guttapercha in all cases where the canals have been treated which involve the periapical tissues. I believe the future will prove this to be necessary in all cases where the infection has involved the periapical tissues.

Referring to the method which Dr. Coolidge mentioned in filling root canals, as advocated by Dr. Callahan, I have this to say: I see no advantage in the addition of rosin in the chloroform. Callahan adds rosin to chloroform, making a thin solution which he introduces into the canal, and with which he makes chloropercha in the canal by pumping the guttapercha point up and down until the outer surface is well dissolved. This chloropercha has the rosin incorporated in it, and Callahan believes he can fill the mouths of the tubuli and all multiple foramina with this resinous guttapercha. He can do so if the tubuli and foramina are open and free from moisture. Callahan shows beautiful specimens of dissected teeth where the canals have thus been filled *out of the mouth.* In the mouth where it is difficult to keep the apical end of the canal absolutely dry, even if we were justified in desiccating the tooth-structure to this extent, which is questionable, I do not believe such beautiful specimens could be shown. Then, too, if we are justified in following Rhein, as I am strongly beginning to believe we are where the periapical tissues have been involved in the infection, it is best to encapsulate the end with plain guttapercha rather than have a substance like rosin incorporated with it.—Dr. I. P. Buckley, in *Dental Review*.

*Iodin Lotion* The most difficult cases we have to contend with are those in which the mucous membrane of the gums and lips are red, inflamed and swollen. The mucus and saliva are thick, ropy and acid. The gums festoons bleed easily. Cervical cavities appear at the necks of all of the anterior teeth. The cavities are so sensitive that they can not be excavated. Begin the treatment by using iodoglycerol:

Zinc iodid, 15 parts; distilled water, 10 parts; iodin crystals, 25 parts; glycerin, 50 parts.

Apply this upon the teeth and gums and saturate the cavities of the teeth every day with an applicator for one, two or three weeks, until the cavities cease to be sensitive, the mouth is clean and all germs are destroyed and the gums receded to nearly or quite normal. At the same time, let the patient use the following gum wash three times each day:

Zinc sulphocarbolate gr. 60; alcohol oz., 1; distilled water oz. 2; true oil of wintergreen gtts. 8.

The sulphocarbolate was introduced to the profession some years ago by Dr. W. H. Whitslar. There is nothing that will contract the gums and cleanse the mouth better than this for a gum wash. The gum wash must be applied with a stiff gum massage brush that will reach the festoons between all the teeth. The dentist should direct the patient's movements at least twice a week for a time to see that the work is properly performed until the gums are pink and hard. At each visit an application of iodo-glycerol must be made also.

The patient must forget the taste of these preparations, since the results are so satisfactory. In the course of a week or two, if the mouth and teeth have been properly cared for, they will be in a condition for the preparation of cavities and filling of the teeth. It is very rare that a pain-obtunder is required after this method of treatment. The cavities may be filled with any material required.

After the mouth has once been placed in a healthy condition the patient must return every three months for further instructions and treatments with iodin.—Dr. Eugene S. Talbot, in *Oral Hygiene.*

*Toxemia from Root Infection* It is not the question of joints, or endocarditis, or thyroiditis, or appendicitis, or gastric ulcer, or nephritis, and all those other things; but the great question is the patient's vitality. Those patients who are suffering with what is known as lymphocytosis show a tremendous increase in the lymphocytes, the white blood corpuscles. That means these patients are calling on their secondary defenses. They have broken through the first line of defenses, which is in the tissue immediately around the teeth. That is the condition of many people. They are not suffering with endocarditis, or other things, but from a toxemia—a chemical poison generated from the bacteria and forced into the system. The vitality becomes lowered to such an extent that the least depression overpowers the resistance. The first line gives way, and the bacteria are then admitted into the system, you get an attack of endocarditis, or other septic infection and your patient dies with an acute disease, although the real sickness from which they have suffered has been of long standing—a toxemia which is going on because of absorption from the mouth.—Dr. Haskins, in *Journal of Allied Societies.*

